# Does the interplay of emotion-related personality traits and reproductive hormones predict individual variation in emotion recognition?

**DOI:** 10.1371/journal.pone.0295176

**Published:** 2023-12-20

**Authors:** Yasaman Rafiee, Charlotte Heine, Anne Schacht

**Affiliations:** 1 Department for Cognition, Emotion and Behavior, Affective Neuroscience and Psychophysiology Laboratory, Institute of Psychology, University of Göttingen, Göttingen, Germany; 2 Leibniz ScienceCampus “Primate Cognition”, Göttingen, Germany; University of Bucharest, Faculty of Biology, ROMANIA

## Abstract

Person-related variation has been identified in many socio-cognitive domains, and there is evidence for links between certain personality traits and individual emotion recognition. Some studies, utilizing the menstrual cycle as a hormonal model, attempted to demonstrate that hormonal fluctuations could predict variations in emotion recognition, but with merely inconsistent findings. Remarkably, the interplay between hormone fluctuations and other person-related factors that could potentially influence emotion recognition remains understudied. In the current study, we examined if the interactions of emotion-related personality traits, namely openness, extraversion, and neuroticism, and the ovulatory cycle predict individual variation in facial emotion recognition in healthy naturally cycling women. We collected salivary ovarian hormones measures from *N* = 129 (*n* = 72 validated via LH test) women across their late follicular and mid-luteal phases of the ovulatory cycle. The results revealed a negative association between neuroticism scores and emotion recognition when progesterone levels (within-subject) were elevated. However, the results did not indicate a significant moderating influence of neuroticism, openness, and extraversion on emotion recognition across phases (late follicular vs. mid-luteal) of the menstrual cycle. Additionally, there was no significant interaction between openness or extraversion and ovarian hormone levels in predicting facial emotion recognition. The current study suggests future lines of research to compare these findings in a clinical setting, as both neuroticism and ovarian hormone dysregulation are associated with some psychiatric disorders such as premenstrual dysphoric disorder (PMDD).

## 1. Introduction

### 1.1 Individual differences in cognitive capacities

Individual variations have been observed in many cognitive domains, including emotion recognition [1], which is the ability to decode the emotional states of others presented through facial, vocal, and body expressions, and therefore contributes significantly to interpersonal communications [2]. While some person-related factors have been identified to be associated with individual variation in emotion recognition (e.g., [3]), the specific determinants of individual variation in this domain and their potential interactions are not yet fully understood, revealing incomplete and inconsistent evidence.

#### 1.1.1 Person-related factors relevant to emotion recognition

A factor proposed to contribute to individual variation in emotion recognition is personality. Personality is defined as “a dynamic organization, inside the person, of psychophysical systems that create the person’s characteristic patterns of behavior, thoughts, and feelings” [4, p. 5]. One of the most recognized models to assess personality is the *Big Five*, including the traits extraversion, neuroticism, agreeableness, conscientiousness, and openness to experience [5, 6]. The *Big Five* model posits that the majority of traits and their descriptions can be classified in five broad domains [7].

The relationship between personality traits and emotion recognition has been investigated in a limited number of studies with inconsistent results. Some evidence supports positive associations between emotion recognition and both openness [8, 9] and extraversion [8, 10], while neuroticism has been linked to both poorer [8] and better [11] recognition of facial emotional expressions. Individuals with high openness scores have been found to be more receptive to environmental stimuli, including emotional states and expressions of others [8]. Similarly, the personality-relationship transaction framework [12] suggests that extraverted individuals tend to be more social and have broader social contacts, making it more likely that those with high openness and extraversion are better at recognizing emotions. Individuals with higher neuroticism scores may exhibit impaired emotion recognition potentially stemming from their tendency to experience negative emotions that might induce avoidance of recognizing emotional states of others [8].

#### 1.1.2 Hormones and emotion recognition

Another person-related factor related to individual variations in emotion recognition is levels of neurochemicals such as hormones. Hormones are widely recognized as biomarkers of individual differences in both behavior and cognition [6]. Studies have been conducted to explore the link between hormone levels (endogenous and exogenous) and emotion recognition ability; however, inconsistent results emerged. Different hormones, such as cortisol [13], oxytocin [14–16], the interplay of cortisol and testosterone [17], and ovarian hormones, including oral contraceptives [18–23], have been investigated in relation to emotion recognition.

Overall, research indicates that both inter-individual and intra-individual differences in hormone levels could explain individual variation in emotion recognition [24, 25]. One hormonal model that accounts for both inter- and intraindividual hormonal levels, is the menstrual cycle. The considerable fluctuation of ovarian hormones, namely estradiol and progesterone, across the menstrual cycle, has recently stimulated noticeable research interest in studying the relationship between the menstrual cycle and socio-cognitive functions [26]. The menstrual cycle consists of follicular and luteal phases [27], with the late follicular phase around the ovulation exhibiting the highest estradiol levels and the mid-luteal phase containing the highest progesterone levels [28].

Studying the conceivable inter- and intra-individual variations in emotion recognition associated with the menstrual cycle is a recent and under-researched field that revealed limited and inconsistent findings due to a lack of standardization in research methodologies. Some studies indicate emotion recognition to be improved in the late-follicular phase (high estradiol) and impaired in the mid-luteal phase (high progesterone; for an overview see [24, 29]). In contrast, other studies did not provide evidence for alteration in emotion recognition across the menstrual cycle (e.g., [21, 30–34]). It is important to note that inconsistencies in the literature arise not only from the use of various methodologies, such as within- versus between-subject comparisons, divergent approaches to cycle phase estimation such as self-report or LH confirmation, and hormonal measurements (see [35]), but also from the lack of consideration of combined influence of different sources of individual differences in emotion recognition.

### 1.2 Study rationale

Women’s unique personality dispositions may influence their emotional responses during their menstrual cycle [36]. Previous studies have focused on either investigating personality traits or menstrual cycle phases (associated with ovarian hormones’ fluctuation) and their links to emotion recognition, while their potential interplay has been neglected so far. In this study, we sought to investigate the potential impact of emotion-related personality traits—including openness, extraversion, and neuroticism—as well as menstrual cycle phases and associated ovarian hormones on the prediction of facial emotion recognition in women. Through exploring the interaction between these factors, we aimed to better understand their role in inter- and intra-individual hormonal variation and overall emotional recognition abilities.

Considering the largest likelihood of conception in the late follicular phase, successful social interaction, requiring fast and accurate emotion recognition, might increase the chance of mating and maximizing reproductive success (see [37]). Individuals with extraversion or openness traits have been assumed to have higher mating opportunities (see [38]), due to their tendencies to seek novelty in the environment and having more social contacts. Therefore, from an adaptive point of view it appears reasonable that individuals exhibit enhanced emotion recognition during the late follicular phase, compared to the mid-luteal phase in these individuals. This effect may be related to elevated levels of estradiol in the late follicular phase.

With regard to openness and extraversion, we hypothesized:

**H1a**) Individuals with higher score in openness have better emotion recognition performance in the late-follicular phase compared to the mid-luteal phase (openness × phase).
**H1b**) Higher scores in openness and higher levels of estradiol (within- and between-subject) is related to improved emotion recognition (openness × estradiol).
**H2a**) Individuals with higher score in extraversion have better emotion recognition performance in the late-follicular phase compared to the mid-luteal phase (extraversion × phase).
**H2b**) Higher scores in extraversion and higher levels of estradiol (within- and between-subject) is related to improved emotion recognition (extraversion × estradiol).

The mid-luteal phase is related to the highest levels of progesterone, which is correlated with negative mood [39] and claimed to increase responses to negative emotions (e.g., [19, 40, 41]). Based on these assumptions, the "window of vulnerability model" suggests that ovarian hormone levels in the luteal phase enhance stress-related physiological and neural reactions, such as endocrine stress responsiveness, memory for affective experiences, and connectivity between the brain’s default mode and salience networks, most commonly seen in affective disorders [42]. This association between high progesterone levels and improved recognition performance in negative expressions of emotion has not been fully replicated (e.g., [32]). It should be noted, however, that a heightened responsiveness to emotional stimuli does not necessarily imply an improved recognition performance. Additionally, a recent well-established study contradicts the "window of vulnerability model," as it indicates no increase in affective symptoms during the mid-luteal phase [43].

Neuroticism is associated with stress and vigilance [38] and has been demonstrated to be negatively related to emotion recognition [8]. The lack of consideration for the moderating effect of neuroticism on the relationship between menstrual cycle phase and emotion recognition has potentially contributed to the inconsistent results in previous studies. While there is ongoing debate and mixed evidence on the relationship between subjective emotional experience and the ability to decode others’ emotions [e.g. 44–47], it is important to note that existing evidence on the association between neuroticism and menstrual cycle phases (and thus ovarian hormone levels) with emotion processing focused primarily on studies examining subjective emotional experiences, such as emotional arousal [48], emotion regulation [e.g., 35], and emotional eating disorders (e.g., binge eating) [49]. Evidence from psychophysiological studies suggests that women exhibiting high levels of neuroticism show increased neural activity (Late Positive Potential–LPP) in response to emotional stimuli during the luteal phase compared to the follicular phase [48]. Additionally, these women showed heightened arousal levels, as indicated by galvanic skin response and heart rate measures, during different phases of their menstrual cycle [50]. These findings raised the question whether the assumed association between the luteal phase, characterized by high levels of progesterone, and emotion recognition tended to be dependent on personality traits, particularly neuroticism. Consequently, additional research is necessary to investigate the link between the moderating effect of neuroticism on the association between cycle phase and emotion recognition.

To bridge the existing gap in the literature, we tested the following hypotheses:

**H3a**) Individuals with higher score in neuroticism have impaired emotion recognition performance in the mid-luteal phase compared to the late-follicular phase (phase × neuroticism).
**H3b**) Higher scores in neuroticism and higher levels of progesterone (within- and between-subject) are related to impaired emotion recognition (progesterone × neuroticism).

Due to the lack of prior evidence regarding other personality traits and emotion recognition, we did not hypothesize about consciousness and agreeableness.

## 2. Methods

This study was conducted as part of a larger project that was preregistered with the Open Science Framework (https://osf.io/dkpf5/). It is worth noting that while the methodology for this study was preregistered as part of the larger data collection process, the formulation of research questions and hypotheses occurred after preregistration. As a result, these particular elements of the study are exploratory in nature.

The study was approved by the local ethics committee of the Institute of Psychology at the University of Göttingen prior to data collection. Following the Declaration of Helsinki (DoH) for human experimentation, all participants acknowledged to take part in the experiment by signing written consent forms. Each participant received either course credit or monetary rewards (25 €) as compensation. The data was collected at the Affective Neuroscience and Psychophysiology laboratory, University of Göttingen.

### 2.1 Participants

One-hundred-eighty women were recruited in the initial data collection; however, the final sample consisted of *N* = 129 (*M*_*Age*_ = 24.1 in years, *SD*_*Age*_ = 3.49, *Range*_*Age*_ = 18–35) women, who completed the experiment. Included participants were healthy, heterosexual, and German native speakers, who had a natural and regular menstrual cycle (*Cycle length* = 25 to 35 days, *M*_*Cycle length*_ = 28.81, *SD*_*Cycle length*_ = 2.02) for at least three months prior to their participation in the study. Fifty-one participants were excluded from the study, primarily because they voluntarily withdrew due to personal reasons like lack of time or interest (44%). Other exclusion factors were an irregular menstrual cycle (32%) or the use of hormonal contraceptives (10%); in addition, we had to exclude two participants due to missing data in the personality questionnaire. Further details can be found in (32). In the current study, *n* = 59 participants reported as single, *n* = 62 were in a relationship, two participants were engaged, three were married, and three reported to be in different forms of relationships. Note that two participants changed their relationship status from the first testing session to the second testing session, from “single” into “in an open relationship” (one participant), and from “other” into “in an open relationship” (one participant). One-hundred-seventeen participants were reported as righthanded.

### 2.2 Study design and procedure

The study was longitudinal and designed to include three sessions. In the first (introductory) session, the eligibility of participants was assessed according to the inclusion criteria (for more details see [32]). Next, participants filled out personality (BFI-2, [51]), empathy (MET, [52]), and motives (MMG, [53]) questionnaires (MET and MMG questionnaires were not in the scope of this study). Moreover, participants were instructed to use highly sensitive (10mIU/ml) urine ovulation test strips from Runbio Biotech Co., Ltd. in order to validate the next fertile phase of their menstrual cycle. They were asked to use the LH strips as soon as their menstruation ended until they saw positive results and send us the pictures of LH tests voluntary. LH tests were asked to use between 10.00 am to 8.00 pm to control for the possible diurnal factors.

The emotion recognition task took place in two separate sessions that were scheduled on the estimated dates of the late follicular and mid-luteal phases of the menstrual cycle, based on backward-counting methods (see [54, 55]). The late follicular phase was estimated as reverse cycle days 16–18, with reverse cycle day 16 as the most ideal date. The mid-luteal phase was considered reverse cycle days 4 to 11, with ideal days as days 6 to 8 [54]. In each session, upon arrival participants first completed a computer-based screening questionnaire concerning their health status and saliva sampling (adapted from [54, 56], and the PANAS mood questionnaire [57] (The mood questionnaire was out of the scope of the current study). As the next step, their saliva samples were collected via passive drooling into salicaps and were immediately stored in a fridge in -80 C. To control for the carry-over effect between sessions two and three, we randomized the menstrual cycle phases between two sessions. Consequently, *n* = 63 women started session two in their late-follicular phase and *n* = 66 started their second session in their mid-luteal phase. The intervals between the two testing sessions were 19.57 days on average (*SD* = 14.14). Due to possible diurnal fluctuations hormones [58, 59] we scheduled session two and three between 12.00 to 4.00 pm and most of the participants were tested at the same time of the day in both session (see [32]).

### 2.3 Study power

To achieve 80% statistical power to detect a medium-sized effect (*d* = .5) in a within-subject study including two sessions across the menstrual cycle (fertile vs. non-fertile) and validation of the fertile phase with an LH test, a sample size of *N* = 48 participants was required (60). Our sample size (*N* = 129, *n* = 72 validated fertile phases via LH tests) is sufficient for statistical analysis of the assumed main effect of the menstrual cycle shift, to detect small to medium-sized effects (see [32]).

### 2.4 Measures

#### 2.4.1 Assessment of the menstrual cycle and ovarian hormones

In the introductory session, we estimated the next day of menses based on the three last dates of participants’ menstruation. The estimated date of ovulation was determined using the backward-counting method. Based on these estimations, each participant took part in the experiments in their late-follicular and mid-luteal phases of the menstrual cycle. The late-follicular phase was validated via LH urine test. Due to the peak of estradiol in the late-follicular and progesterone’s peak in the mid-luteal phases, we decided to study these two phases (see [60, 61]).

The levels of estradiol (E2) and progesterone (P4) were measured in saliva during each phase of the menstrual cycle using Chemiluminescence Immunoassays at the Endocrinology Laboratory of the Technical University of Dresden. The samples were analyzed as a single determination, which is less accurate than duplicate determination, and the reported coefficient of variation was below 10%. Furthermore, as an external validation of hormonal measures, we conducted a regression analysis of the estradiol/progesterone ratio (E/P) and cycle phases, revealing a significant association between hormones level and cycle phases (β = 0.114, SE = 0.000, 95% CI = [0.11; 0.12], t = 130.3, *p* < .001).

Additionally, to investigate if levels of ovarian hormones fluctuate significantly across the late follicular and mid-luteal phases of the menstrual cycle, we fitted two linear mixed models for each hormone. Each model included one hormone (log-transformed) as the outcome variable, cycle phase as the predictor, and participant ID as the random effect. The model with estradiol as the outcome revealed a slight but significant reduction in estradiol levels in the mid-luteal phase compared to the late follicular phase (β = -0.09, SE = 0.002, 95% CI = [-0.09; 0.10], *p* < .001), and the model with progesterone as the outcome showed a significant and strong rise of the progesterone levels in the mid-luteal phase compared to the late follicular phase (β = 0.73, SE = 0.002, 95% CI = 0.72; 0.73], *p* < .001).

We excluded hormone outliers ± 3 SDs from the sample mean (see [62]). Five hormonal measures including three estradiol and two progesterone measures were excluded from the data.

#### 2.4.2 Assessment of personality measures

Personality traits were assessed using the German version of the Big-Five Inventory (BFI-2), consisting of 60 sentences [51]. The BFI-2 measures five major dimensions of personality: openness, conscientiousness, extraversion, agreeableness, and neuroticism. Extraverts are described as being outgoing, optimistic and enjoying social contacts, whereas neuroticism is associated with stress and a predisposition to worry [5, 63]. Openness is often characterized as having a heightened sensitivity to aesthetics and being intellectually curious, attentive to inner feeling, and imaginative [9, 64]. Conscientiousness is linked to traits as reliability, hard work and goal-directedness [8, 64]. Individuals with high agreeableness are recognized for their empathetic nature toward others, typically exhibiting characteristic such as compassion and politeness [65].

Participants provided their answers to each sentence on a Likert scale ranging from one to five (1 = strongly disagree, 2 = somewhat disagree, 3 = neither agree nor disagree, 4 = somewhat agree, 5 = strongly agree). Each personality dimension consisted of three subcategories (facets), each represented by four sentences. Personality trait scores were calculated by averaging the values selected for each personality trait and its associated facets [51].

#### 2.4.3 Emotion recognition task

The emotion recognition task was adapted from [17] and consisted of three separate blocks of stimuli from the visual, auditory, or audiovisual modality, respectively. This study solely concentrated on facial expressions since the pertinent literature available pertains exclusively to this domain. Stimuli consisted of 24 face portraits for each of six expressions (anger, disgust, sadness, fear, happiness, neutral), with equal numbers of male and female faces, selected from the Radboud face database [66], and matched in their luminance [see 17]. The emotion recognition task was used to measure emotion recognition accuracy and reaction times; however, there was no time limitation for the responses to the trials.

To familiarize participants with the experimental procedure, the task began with three practice trials. All trials started with a blank screen for 1000 ms, followed by a fixation cross in the center of the screen for 1000 ms. Afterwards, the target stimulus, varying in duration between 319 and 4821 ms, appeared in the center of the screen. Subsequently, a circular multiple-choice answer display with labelled emotion categories was presented in the center of the screen, along with a selection cursor. The arrangement of emotion labels within this circle was counterbalanced but remained constant for each participant. Participants were instructed to select the corresponding emotion label as accurately and quickly as possible using the mouse to move the cursor to the certain label. Correct responses were considered as hits (see [17, 32]).

### 2.5 Statistical analysis

To test our hypothesis, the data was analyzed using Generalized Linear Mixed Models (GLMMs; [67]) with binomial error structure and logit link function implemented in R Software version 4.0.3 and R studio version 1.4.1106. In each model, emotion recognition accuracy (as a binomial variable: correct vs. false) was the outcome variable, session number served as the control variable, and subject ID as the random intercept. In models investigating the interaction of ovulatory cycle and personality traits, the reference category for the ovulatory cycle was determined as the mid-luteal phase. Standard p-value 0.05 was determined as the cut-off criterion for two-tailed distributions.

Prior to including variables in the model, we checked the distribution of hormone and personality measures using visual inspection and the Shapiro-Wilk test. As expected, both hormone distributions significantly deviated from normality (estradiol: W = 0.90, p < 0.01, progesterone: W = 0.83, p < 0.01). Additionally, we assessed the distribution of neuroticism scores, which seemed to follow the normal distribution (neuroticism: W = .99, p = .809). In contrast, distributions of both openness and extraversion scores were found to be non-normal (openness: W = 0.97, p = .01; extraversion: W = 0.96, p = .004). Importantly, upon visual inspection of the histograms for openness and extraversion, significant skewness was not observed.

To track within-subject fluctuations of hormonal measures across the late follicular and mid-luteal phases (following [62]), the measures were subject mean-centered and then scaled by dividing them by a constant (see [32]). This resulted in values ranging from −.5 to .5, which simplifies calculation in the linear mixed model. Subject mean centering was employed to isolate the influence of the within- and between-subject variation of hormones [68]. To create the between-subject hormone variable, we computed the mean of each hormone measure across two phases and subsequently log-transformed the between-subject levels of estradiol and progesterone to achieve normal distribution. To facilitate the interpretation of model outcome, log-transformed between-subject levels of ovarian hormones and personality scores were z-transformed (see [69]).

For each model, we assessed Variance Inflation Factors (VIF; [70]) with a model lacking the interaction term. No collinearity issue was found for each model (maximum VIF: 1.03). To evaluate the goodness of fit of the fitted model, the log-likelihood function of the fitted model was compared with the log-likelihood function of the minimal (reduced) model lacking the predictor or the interaction of interest (see [71]). Model stability was good for all models.

The following packages were used: ggplot2 3.3.3 [72], and sjplot 2.8.7 [73] for data visualization, lme4 1.1.26 [74] for computing models, and car 3.0.10 [75] for assessing collinearity within predictors.

## 3. Results

### 3.1 Descriptive analysis

#### 3.1.1 Emotion recognition

The average proportion of hits in facial emotion recognition was *M*_*accuracy*_ = 0.912 (*SD* = 0.282) in the late follicular and *M*_*accuracy*_ = 0.913 (*SD* = 0.281) in the mid-luteal phase. The average reactions times for facial emotion recognition was *M*_*reaction times*_ = 1.29 s (*SD* = 0.90) in the late follicular phase and *M*_*reaction time*_ = 1.28 s (*SD* = 1.65) in the mid-luteal phase.

#### 3.1.2 Personality

The average score for personality traits was as following: *M*_*agreeableness*_ = 3.96 (*SD* = 0.65), *M*_*consciousness*_ = 3.45 (*SD* = 0.75), *M*_*extraversion*_ = 3.45 (*SD* = 0.75), *M*_*openness*_ = 3.75 (*SD* = 0.75), M_neuroticism_ = 2.81 (*SD* = 0.67).

#### 3.1.3 Hormone measures

The average levels of estradiol excluding outliers (±3 SDs) in the late follicular phase was *M*_*Estradiol*_ = 6.70 (*SD* = 4.46, range = 1.05 to 21.76) pg/mL and the average progesterone levels was *M*_*Progesterone*_ = 39.64 (*SD* = 25.27, range = 14.60 to 218.16) pg/mL. In the mid-luteal phase, the average of estradiol levels excluding outliers (±3 SDs) was *M*_*Estradiol*_ = 6.09 (*SD = 3*.*82*, range = 0.70–19.62) pg/mL and the average of progesterone levels was *M*_*Progesterone*_ = 84.13 (SD = 45.65, range = 20.18–266.16) pg/mL.

#### 3.1.4 Cycle phase × openness model (H1a)

In the first hypothesis, we assumed an interacting effect of the menstrual cycle (late-follicular vs. mid-luteal) and openness on emotion recognition. A GLMM was fitted, with cycle phase (confirmed by LH test, *n* = 72) and openness scores (scaled) as fixed effects, and their interaction. The model revealed no significant interaction of cycle phase and openness in predicting facial emotion recognition (β = 0.033, SE = 0.05, 95% CI = [0.93–1.14], z = 0.63, OR = 1.03, *p* = .530; see Table 1). Comparison between the log-likelihood of the main model with the log-likelihood of the model lacking the interaction effect (reduced model) revealed no significant differences between two models (χ2 = 0.39, df = 1, *p* = .532). The model outcome showed that women performed better in the second session compared to the first session. We ran an additional model, including menstrual cycle phase–regardless of LH results–(*N* = 129) to control for the robustness of our findings. The additional model showed no significant interaction of phase and openness on emotion recognition (β = -0.016, SE = 0.04, 95% CI = [0.91–1.06], z = -0.418, OR = 0.98, *p* = .676) (See supporting information–S1 Table).

**Table 1:** Results of Generalized Linear Mixed Model (GLMM) testing the interaction of menstrual cycle phase (late-follicular vs. mid-luteal) and personality traits on facial emotion recognition (n = 72).

	*Estimates*	*SE*	*z*	*p*	*OR*	*95% CI*
**Openness**						
Phase [mid-luteal]	0.070	0.052	1.332	0.183	1.07	0.97–1.19
Openness	0.002	0.066	0.030.	0.976	1.00	0.88–1.14
Phase × Openness	0.033	0.052	0.628	0.530	1.03	0.93–1.14
Session	0.272	0.052	5.191	**<0.001**	1.31	1.18–1.45
**Extraversion**						
Phase [mid-luteal]	0.065	0.052	1.243	0.214	1.07	0.96–1.18
Extraversion	0.035	0.065	0.530	0.596	1.04	0.91–1.18
Phase × Extraversion	-0.083	0.049	-1.690	0.091	0.92	0.84–1.01
Session	0.291	0.053	5.476	**<0.001**	1.34	1.21–1.48
**Neuroticism**						
Phase [mid-luteal]	0.066	0.052	1.258	0.208	1.07	0.96–1.18
Neuroticism	-0.181	0.067	-2.696	**0.007**	0.83	0.73–0.95
Phase × Neuroticism	0.064	0.052	1.224	0.221	1.07	0.96–1.18
Session	0.268	0.052	5.119	**<0.001**	1.31	1.18–1.45

#### 3.1.5 Estradiol × openness model (H1b)

We fitted a GLMM, including levels of estradiol (within- and between subject), scores of openness, and their interaction. The model indicated no significant interacting effect between estradiol levels (neither within- nor between-subject) and openness scores (see Table 2). Women performed better in the second than in the first session, independent of the hormone levels. Comparing the log-likelihood of the reduced model (lacking the interaction term) with the log-likelihood of main model indicated no significant differences in predicting the outcome variable (χ2 = 2.56, df = 2, *p* = 0.277).

**Table 2:** Results of the Generalized Linear Mixed Model (GLMM), testing interaction of ovarian hormones and personality traits on emotion recognition (N = 129).

	*Estimates*	*SE*	*z*	*P*	*OR*	*95% CI*
**Estradiol × Openness**						
Estradiol (within-subject)	-0.033	0.142	-0.233	0.816	0.97	0.73–1.28
Estradiol (between-subject)	0.051	0.050	1.004	0.315	1.05	0.95–1.16
Openness	-0.020	0.051	-0.398	0.691	0.98	0.89–1.08
Estradiol (within-subject) × Openness	-0.196	0.136	-1.437	0.151	0.82	0.63–1.07
Estradiol (between-subject) × Openness	-0.041	0.056	-0.730	0.466	0.96	0.86–1.07
Session	0.270	0.038	7.133	**<0.001**	1.31	1.22–1.41
**Estradiol × Extraversion**						
Estradiol (within-subject)	0.006	0.141	0.041	0.967	1.01	0.76–1.33
Estradiol (between-subject)	0.056	0.050	1.133	0.257	1.06	0.96–1.17
Extraversion	-0.037	0.050	-0.747	0.455	0.96	0.87–1.06
Estradiol (within-subject) × Extraversion	-0.284	0.139	-2.036	**0.042**	0.75	0.57–0.99
Estradiol (between-subject) × Extraversion	0.016	0.053	0.297	0.766	1.02	0.92–1.13
Session	0.269	0.038	7.115	**<0.001**	1.31	1.21–1.41
**Progesterone × Neuroticism**						
Progesterone (within-subject)	-0.142	0.149	-0.958	0.338	0.87	0.65–1.16
Progesterone (between-subject)	-0.039	0.049	-0.787	0.432	0.96	0.87–1.06
Neuroticism	-0.106	0.048	-2.188	**0.029**	0.90	0.82–0.99
Progesterone (within-subject) × Neuroticism	-0.284	0.138	-2.061	**0.039**	0.75	0.57–0.99
Progesterone (between-subject) × Neuroticism	-0.088	0.046	-1.902	0.057	0.92	0.84–1.00
Session	0.263	0.038	6.935	**<0.001**	1.30	1.21–1.40

#### 3.1.6 Cycle phase × extraversion (H2a)

The GLMM, including cycle phase, extraversion scores, and their interaction indicated no significant interaction of cycle phase and extraversion (β = -0.83, SE = 0.049, 95% CI = [0.84–1.01], z = -1.690, OR = 0.92, *p* = .091; Table 1). Comparison between the log-likelihood of the main model with the log-likelihood of the reduced model (lacking the interaction term) revealed no significant difference in predicting the outcome variable (χ2 = 2.836, df = 1, *p* = .092).

To test for robustness of the results, we fitted an extra model including cycle phase, regardless of LH confirmation (*N* = 129), extraversion, their interaction, session number and Subject ID. The model revealed consistent non-significant results (β = -0.031, SE = 0.037, 95% CI = [0.90–1.04], z = -0.820, OR = 0.97, *p* = .412; see supporting information–S1 Table).

#### 3.1.7 Estradiol × extraversion model (H2b)

The GLMM including estradiol (within- and between-subject), extraversion, and their interaction revealed no significant interaction between estradiol levels (neither within- nor between-subject) and extraversion scores (see Table 2). Moreover, comparing the log-likelihood of the main model with the log-likelihood of the reduced model (lacking the interaction term) revealed no significant differences in predicting the outcome variable (χ2 = 4.2, df = 2, *p* = 0.122).

#### 3.1.8 Cycle phase × neuroticism (H3a)

A GLMM model was fitted to assess the interaction between menstrual cycle phases (late-follicular vs mid-luteal, confirmed via LH) and neuroticism on emotion recognition. The results showed no significant interaction effect of cycle phase and neuroticism score in facial emotion recognition (β = 0.064, SE = 0.052, 95% CI = [0.96–1.18], z = 1.224, OR = 1.07, *p* = .221). The log-likelihood ratio test revealed no significant differences between the main and reduced models (χ2 = 1.48, df = 1, *p* = .222, see Table 1). Women performed better in the second session than in the first session.

We fitted an additional model, including the menstrual cycle phase regardless of LH results (*N* = 129) as robustness check. The model confirmed the non-significant interacting effect of phase and neuroticism on emotion recognition accuracy (β = 0.062, SE = 0.037, 95% CI = [0.99–1.14], z = 1.662, OR = 1.06, *p* = .097; see supporting information, S1 Table).

#### 3.1.9 Progesterone × neuroticism model (H3b)

**Fig 1:**
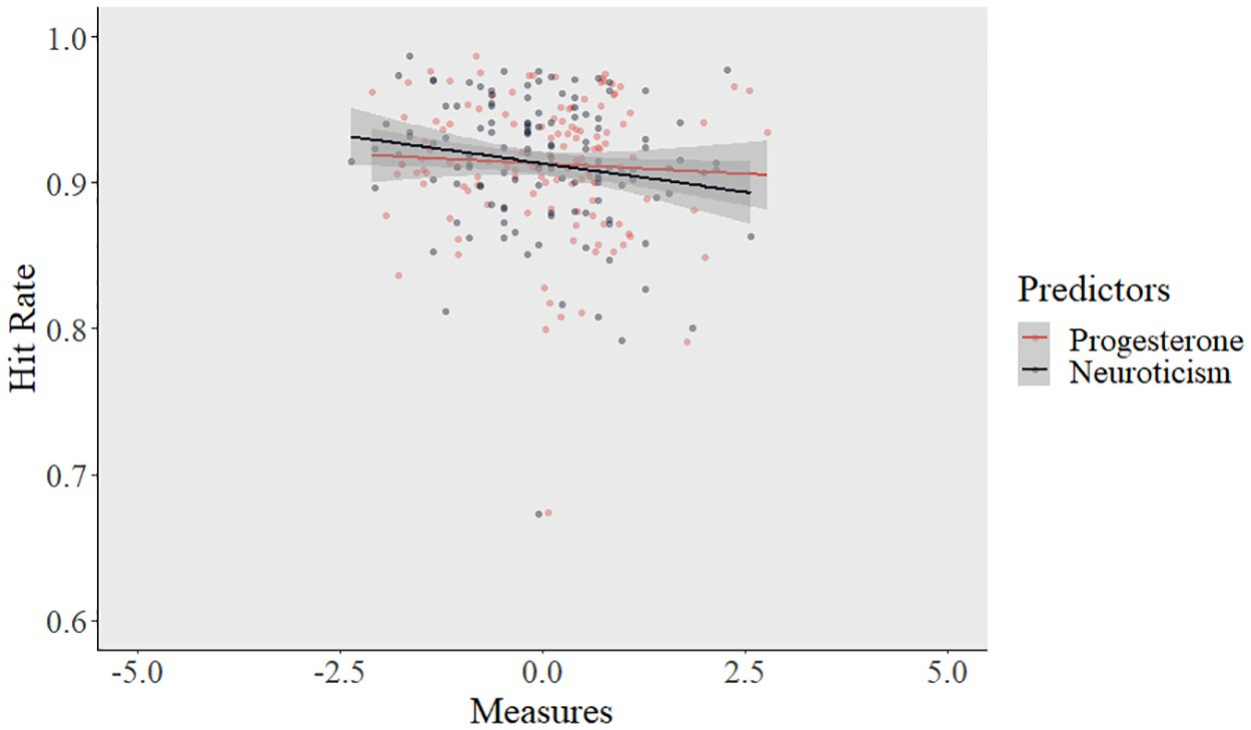
Elevated progesterone levels in individuals with higher neuroticism score decreases emotion recognition performance. Orange line shows the changes in emotion recognition accuracy (hits) as the function of neuroticism scores. Blue line shows the changes in emotion recognition accuracy (hits) as the function of progesterone levels (between-subject). Note: Both progesterone and neuroticism measures were scaled for plotting purposes. Between-subject progesterone measures were averaged, then log-transformed and then scaled.

Next, we tested the potential interaction of progesterone levels and neuroticism by fitting a GLMM, including progesterone levels (within- and between-subject), neuroticism, and their interaction. The model revealed a significant interaction of progesterone (within- subject) **×** neuroticism (β = -0.284, SE = 0.137, 95% CI = [0.58–0.99], z = -2.065, OR = 0.75, *p* = .039; see Table 2). Aligned with our prediction, increasing levels of progesterone (within-subjects) in individuals with higher neuroticism scores is associated with significantly impaired emotion recognition (Fig 1). The likelihood ratio test showed that the main model predicted the outcome significantly better than the reduced model (χ2 = 7.78, df = 2, *p* = .020).

### 3.2 Exploratory analysis

We conducted an exploratory analysis to examine the three-way interaction between ovulatory cycle, personality traits, and emotion category in predicting facial emotion recognition. Additionally, we repeated the analysis using estradiol and progesterone instead of the ovulatory cycle phases. The results indicated no significant interaction between ovulatory cycle and personality traits in predicting specific facial expression. These findings remained consistent when examining the interaction between ovarian hormone levels and personality traits in relation to specific facial expressions. The script and results can be accessed via this link: https://osf.io/dkpf5/.

## 4. Discussion

The current study presents preliminary evidence of the potential moderating role of emotion-related personality traits in the assumed link between the ovulatory cycle and facial emotion recognition. Our findings indicate that neuroticism was associated with impaired emotion recognition, when progesterone levels were high (within-subjects). Contrary to our predictions, no significant shifts in emotion recognition were found when examining the interactions between the personality traits studied (neuroticism, extraversion, and openness) and cycle phase in predicting facial emotion recognition. Additionally, no evidence was found to support the interactions between both openness and extraversion and ovarian hormones in predicting facial emotion recognition.

Previous research examining the assumed association between the ovulatory cycle–and thus the level of ovarian hormones–and emotion recognition has yielded mixed evidence. Some studies have indicated enhanced emotion recognition during the follicular phase, which is associated with elevated levels of estradiol (e.g. [19, 29, 76, 77]). On the contrary, other studies have not observed this association (e.g. [22, 30, 31, 33, 34]). These heteroginities have been attributed to various methodological factors, such as differences in hormonal assessment, estimation of cycle phase, and experimental paragidms, as well as the lack of methodological rigor, such as low statistical power and cross-sectional comparisons (see [24]).

In a recent study involving the dataset analyzed here (*N* = 131), Rafiee and co-workers [32] examined potential alterations in emotion recognition during ovulatory cycle phases (late follicular vs. mid-luteal) and reported null findings. In contrast to their prediction that emotion recognition would be impaired with elevated progesterone levels, they did not find any supportive evidence. Remarkably, the present study revealed significant changes in emotion recognition with increased progesterone levels (within-subjects) are elevated, after considering the moderating effect of neuroticism. This result may clarify the inconsistent findings in previous literature, emphasizing the importance of potential moderating variables in the relationship between the psycho-endocrine system and emotion recognition. Such approach is further supported by the intricate and interactive impact of reproductive steroid hormones on the body and brain at the molecular, organ, tissue, and behavioral levels [78]. These interaction–studied here in conjunction with personality traits–appear to also manifest in individual differences. A comprehensive understanding of these interactions necessitates a multi-level approach that goes beyond their possible main effects [78].

Aware of the novelty and need for replication, we would like to provide some cautious interpretations of this study’s findings: Despite the potential moderating effect of neuroticism on emotion recognition alteration when progesterone levels are elevated, this topic has been largely understudied. Few studies have examined the joint role of neuroticism and the mid-luteal phase in emotional processing across the menstrual cycle, including (subjective) emotional experiences, emotion regulation, and eating disorders; however, these studies have yielded inconsistent findings (e.g. [36, 48, 50, 79]).

Further evidence for the potential interplay between neuroticism and progesterone in emotion recognition could be obtained through research on premenstrual syndrome (PMS) symptoms during the luteal phase. Studies have indicated that increased levels of neuroticism can intensify PMS symptoms during the luteal phase [80, 81]. PMS affects approximately 80% of women, and it has been suggested that its high frequency may indicate an evolutionary advantage, possibly associated with preventing sexual or relationship breakdowns. However, more research is needed to comprehend its significance [82].

Neuroticism, which is associated with increased vigilance to danger [38], may also serve an adaptive purpose during the luteal phase. PMS is linked to varying individual responses to the fluctuation of ovarian hormones, highlighting the relevance of individual differences in reproductive steroid hormones [78]. The "window of vulnerability" model [42] suggests that the mid-luteal phase of the menstrual cycle, characterized by elevated progesterone levels, increases the vulnerability to negative affective symptoms. However, a recent study by Guevarra et al. [43] found that perceived stress had a greater impact on affective symptoms than the menstrual cycle phase, and found no compelling evidence for an increase in affective symptoms during the mid-luteal phase. The stress perception hypothesis [83] suggests that neuroticism is the main factor behind perceived stress. However, current research on the connection between emotional experience and emotion recognition has mixed findings, as demonstrated in recent studies by Holland et al. [45] and Wearne et al. [47]. Altogether, the combined effect of neuroticism and the luteal phase may further exacerbate the detrimental impact on emotion recognition in women.

Both openness and extraversion have been linked to social behavior, but it was not found to significantly influence emotion recognition with variations across the menstrual cycle in our sample. This null result requires further validation through replication and studies that distinguish the unique contributions of openness and extraversion to emotion recognition. For example, recent research has indicated that individuals with higher levels of extraversion tend to exhibit greater proficiency in emotional encoding compared to emotional decoding [84].

### 4.1 Limitations

There are some limitations to the current study that needs to be acknowledged. One of the limitations of this study is the use of an immunoassay approach to measure salivary estradiol and progesterone. Arslan et al. [85] recommend using the Liquid Chromatography–Mass Spectrometry (LC-MS/MS) method, considered the gold standard for ovarian hormones measurement, for more accurate results in hormonal studies. Therefore, our findings should be interpreted in the light of limitation [32]. This constraint could possibly explain the absence of association between openness or extraversion and estradiol’s interaction on emotion recognition. Another potential limitation of this study that may account for the lack of significant findings is the study’s power. It is important to acknowledge that for detecting interaction effects, as in our current study, a larger sample size may have been required to reliably detect medium-sized effects.

Additionally, this study only included women in the late follicular and mid-luteal phases of their menstrual cycle. It might be necessary to conduct a wider examination of emotion recognition including pre and post menstrual points where hormone levels are at their lowest, to ensure a more valid comparison. Furthermore, although personality is considered to be a trait characteristic, further research may benefit from the reassessment of personality measures across experimental sessions in order to examine potential changes in self-appraisal during the menstrual cycle.

Another limitation could be the context-dependence of the relationship between hormones and behavior. As suggested by Sundin et al. [6], hormone levels such as cortisol and testosterone that are measured in neutral settings may not accurately reflect their role in behavior and cognition. Therefore, future studies should consider context and situational factors when exploring the link between hormones and behavior [6].

We limited our sample to healthy young women of reproductive age, primarily consisting of university students. Future research should examine the moderating effect of personality traits on the relationship between emotion recognition and menstrual cycle in a more diverse and representative population. It would also be interesting for future research to explore the same research questions in women who use contraceptives. Additionally, it would be valuable to compare these findings in a clinical setting, particularly investigating the relationship between neuroticism and emotion recognition in women with Premenstrual Dysphoric Disorder (PMDD) during the luteal phase. Finally, future studies should consider including measures of PMS symptoms, such as pain and stress levels during the luteal phase, as possible predictors of emotion recognition ability during the luteal phase.

## 5. Conclusion

This study provides valuable insight into the joint role of dispositional traits and biological markers (menstrual cycle and hormones) in predicting individual differences in emotion recognition. Understanding these differences in emotion recognition can shed light on the complex interplay of various factors involved in this cognitive ability. Recognizing and appreciating variations in cognitive abilities and their contributing determinants helps to develop novel approaches to understanding cognitive development and performance [86].

## Supporting information

S1 Table Results of Generalized Linear Mixed Model (GLMM) testing the interaction of menstrual cycle phase (late follicular vs. mid-luteal) and personality traits on facial emotion recognition (N = 129). (DOCX)

S1 Checklist STROBE statement—Checklist of items that should be included in reports of observational studies. (DOCX)
