# Supplementary material for: Does the interplay of emotion-related personality traits and reproductive hormones predict individual variation in emotion recognition?
Source: PLoS One. 2023 Dec 20;18(12):e0295176. doi: 10.1371/journal.pone.0295176 (PMC10732445; doi:10.1371/journal.pone.0295176)
Supplement: S1 Table — (DOCX) [file pone.0295176.s001.docx]

**Table S1: Results of Generalized Linear Mixed Model (GLMM) testing the interaction of menstrual cycle phase (late follicular vs. mid-luteal) and personality traits on facial emotion recognition (N =129).**

|  | Estimates | SE | z | p | OR | 95% CI |
| --- | --- | --- | --- | --- | --- | --- |
| **Model Phase × Openness (N = 129)**  Phase [mid-luteal] | -0.002 | 0.037 | -0.063 | 0.950 | 1.00 | 0.93 – 1.07 |
| Openness | -0.023 | 0.054 | -0.438 | 0.661 | 0.98 | 0.88 – 1.08 |
| Phase × Openness | -0.016 | 0.039 | -0.418 | 0.676 | 0.98 | 0.91 – 1.06 |
| Session | 0.266 | 0.038 | 7.077 | **<0.001** | 1.30 | 1.21 – 1.40 |
| **Model Phase × Extraversion (N = 129)**  Phase [mid-luteal] | -0.002 | 0.037 | -0.056 | 0.955 | 1.00 | 0.93 – 1.07 |
| Extraversion | -0.027 | 0.053 | -0.504 | 0.614 | 0.97 | 0.88 – 1.08 |
| Phase × Extraversion | -0.031 | 0.037 | -0.820 | 0.412 | 0.97 | 0.90 – 1.04 |
| Session | 0.268 | 0.038 | 7.120 | **<0.001** | 1.31 | 1.21 – 1.41 |
| **Model Phase × Neuroticism (N = 129)**  Phase [mid-luteal] | -0.008 | 0.037 | -0.201 | 0.841 | 0.99 | 0.92 – 1.07 |
| Neuroticism | -0.131 | 0.052 | -2.527 | **0.012** | 0.88 | 0.79 – 0.97 |
| Phase × Neuroticism | 0.062 | 0.037 | 1.661 | 0.097 | 1.06 | 0.99 – 1.14 |
| Session | 0.264 | 0.037 | 7.071 | **<0.001** | 1.30 | 1.21 – 1.40 |
